# Junctional Adhesion Molecule (JAM)-C Deficient C57BL/6 Mice Develop a Severe Hydrocephalus

**DOI:** 10.1371/journal.pone.0045619

**Published:** 2012-09-18

**Authors:** Lena Wyss, Julia Schäfer, Stefan Liebner, Michel Mittelbronn, Urban Deutsch, Gaby Enzmann, Ralf H. Adams, Michel Aurrand-Lions, Karl H. Plate, Beat A. Imhof, Britta Engelhardt

**Affiliations:** 1 Theodor Kocher Institute, University of Bern, Bern, Switzerland; 2 Edinger Institute, Goethe University Medical School, Frankfurt/Main, Germany; 3 Max Planck Institute for Molecular Biomedicine, Department of Tissue Morphogenesis, Münster, Germany; 4 Faculty of Medicine, University of Münster, Münster, Germany; 5 INSERM, Centre de Recherche en Cancérologie de Marseille, Institut Paoli-Calmettes, Marseille, France; 6 Department of Pathology and Immunology, University of Geneva, CMU, Geneva, Switzerland; UAE University, Faculty of Medicine & Health Sciences, United Arab Emirates

## Abstract

The junctional adhesion molecule (JAM)-C is a widely expressed adhesion molecule regulating cell adhesion, cell polarity and inflammation. JAM-C expression and function in the central nervous system (CNS) has been poorly characterized to date. Here we show that JAM-C^−/−^ mice backcrossed onto the C57BL/6 genetic background developed a severe hydrocephalus. An in depth immunohistochemical study revealed specific immunostaining for JAM-C in vascular endothelial cells in the CNS parenchyma, the meninges and in the choroid plexus of healthy C57BL/6 mice. Additional JAM-C immunostaining was detected on ependymal cells lining the ventricles and on choroid plexus epithelial cells. Despite the presence of hemorrhages in the brains of JAM-C^−/−^ mice, our study demonstrates that development of the hydrocephalus was not due to a vascular function of JAM-C as endothelial re-expression of JAM-C failed to rescue the hydrocephalus phenotype of JAM-C^−/−^ C57BL/6 mice. Evaluation of cerebrospinal fluid (CSF) circulation within the ventricular system of JAM-C^−/−^ mice excluded occlusion of the cerebral aqueduct as the cause of hydrocephalus development but showed the acquisition of a block or reduction of CSF drainage from the lateral to the 3^rd^ ventricle in JAM-C^−/−^ C57BL/6 mice. Taken together, our study suggests that JAM-C^−/−^ C57BL/6 mice model the important role for JAM-C in brain development and CSF homeostasis as recently observed in humans with a loss-of-function mutation in JAM-C.

## Introduction

Junctional adhesion molecules C (JAM-C) (previously referred to as hJAM-3 and mJAM-2) is a type I transmembrane protein and a member of the immunoglobulin (Ig) superfamily. Based on its molecular structure JAM-C has been assigned together with JAM-A, JAM-B, JAM-4, JAM-L, ESAM and CAR to the CTX sub-family of the Ig superfamily. The extracellular domain of JAM-C consists of a membrane distal V_H_ -and a membrane proximal C_2_-type Ig-like domain, a single transmembrane segment and a relatively short cytoplasmic tail with four putative phosphorylation sites and a PDZ-domain binding motif that allows interaction of JAM-C with different scaffolding proteins [1].

JAM-C was orignially described on vascular and lymphatic endothelial cells, where it localizes to cell-cell contacts [2] and on human T cells [3]. Since then expression of human JAM-C has been detected on additional subsets of leukocytes as well as lymphomas and on platelets (summarized in [1]. In contrast, expression of JAM-C in mice was found to be restricted to hematopoietic precursors and is absent on differentiated leukocytes [4], [5]. Furthermore, expression of JAM-C has been described on a wide range of non-hematopoietic cells including endothelial and epithelial cells [6], fibroblasts [7], smooth muscle cells [8] and spermatids [9].

In endothelial and epithelial cells JAM-C localizes to intercellular contacts. With its extracellular domain JAM-C can engage in homophilic binding, adhere to JAM-B and the integrins αMβ2 and αX β2summarized in [1]) and has therefore been assigned an important role in mediating leukocyte migration across endothelial and epithelial barriers in a number of inflammatory settings [4], [10] and during ischemia/reperfusion [11]. Through its cytoplasmic tail JAM-C binds to ZO-1 and PAR-3 suggesting a predominant localization of JAM-C in endothelial and epithelial tight junctions and a possible role in cell polarization [12]. An essential role of JAM-C in cell polarization has in fact been proven by the observation that JAM-C^−/−^ mice that fail to differentiate round spermatids into polarized mature spermatozoa and thus show male infertility [9]. Finally, JAM-C has also been shown to be involved in tumor growth and metastasis [13] [14].

Due to this broad spectrum of biological functions, it is not surprising that JAM-C^−/−^ mice were found to exhibit additional severe phenotypes including growth retardation, megaoesophagus, disturbed neutrophil homeostasis and increased susceptibility to opportunistic infections, resulting in poor survival under conventional housing conditions [15]. Housing of JAM-C^−/−^ mice in ventilated isolaters was found to partially rescue this phenotype confirming an important role of JAM-C in fighting opportunistic infections. Finally, re-introduction of vascular JAM-C expression rescued granulocyte homeostasis and survival of JAM-C^−/−^ mice, emphasizing the importance of endothelial JAM-C in proper immune function [15], [16].

Recent studies have provided evidence for a role of JAM-C in the organization of the peripheral nervous system, where it was found to be expressed in Schwann cells of myelinated peripheral nerves and in perineural cells [17] [18]. In contrast JAM-C expression was described to be absent in the central nervous system (CNS) in some studies [2], [17] but not others [3]. The recent discovery of a homozygous mutation in JAM-C in a large consanguineous family from the United Arab Emirates, members of which developed a rare autosomal-recessive syndrome characterized by severe hemorrhagic destruction of the brain, suggest a novel, previously unrecognized role for JAM-C in maintaining cerebrovascular integrity [19].

To further study the role of JAM-C in the development and maintenance of the blood-brain barrier (BBB) and in neurological disorders *in vivo,* we have backcrossed JAM-C^−/−^ mice into the C57BL/6 background. We found that JAM-C^−/−^ C57BL/6 mice developed a severe hydrocephalus, characterized by extremely enlarged lateral ventricles, cortical thinning and disturbances in CSF circulation. JAM-C expression could be localized to the BBB and choroid plexus endothelial cells and to choroid plexus epithelial cells. Notably, JAM-C was highly expressed in ependymal cells lining the ventricles. Although we found numerous hemorrhages in JAM-C^−/−^ C57BL/6 mice the development of the hydrocephalus was independent of JAM-C expression by endothelial cells, as endothelial cell specific re-expression of JAM-C in JAM-C^−/−^ mice neither rescued the survival nor affected the incidence or severity of the hydrocephalus. Our study therefore suggests an important role for JAM-C in brain development and CSF homeostasis and shows that JAM-C^−/−^ C57BL/6 mice provide a valuable model for the defects recently observed in humans with a loss-of-function mutation in JAM-C [19].

## Materials and Methods

### Mice

Mice were bred in individually ventilated cages (IVC) at SPF conditions according to the animal protection law of the Kanton Bern, Switzerland. Breeding of mice and all animal experiments shown in this study have been approved by the vetenary office of the Kanton Bern under the permission number 48/08. JAM-C deficient mice produced by insertion of an IRES-LacZ-pA cassette and neomycin resistance cassette and transgenic mice with endothelial-specific overepression of JAM-C (pHHNS-JAM-C) referred to as Tie2 JAM-C mice, were described previously [4], [9]. Both lines were backcrossed onto the C57BL/6 background for 8 to 12 generations in our laboratory. Production of JAM-C^−/−^//Tie2 JAM-C C57BL/6 mice was achieved by crossing JAM-C^+/−^ C57BL/6 mice with Tie2 JAM-C C57BL/6 mice resulting in JAM-C^+/−^//Tie2 JAM-C C57BL/6 mice and subsequent crossing with JAM-C^+/−^ C57BL/6 mice.

### Scoring of the Severity of Hydrocephalus Development

Hydrocephalus development was scored as follows: 0 = macroscopically normal phenotype; 1 = normal snout, slightly enlarged skull; 2 = pointed snout, enlarged skull, often untended coat; 3 = extremely pointed snout, extremely enlarged skull, untended coat.

### Genotyping of JAM-C−/− Mice and JAM-C−/− Mice Transgenic for Tie2 JAM-C

Tail biopsies were digested with proteinase K (Merck) to extract genomic DNA. Samples were subsequently subjected to polymerase chain reaction. To identify mice that carry the JAM-C neo allele the following primer pair was used: 5′ -TCATGTGGATAGCCACAACA- 3′ and 5′ -ACATCTGTGCGACCTGCCA- 3′ (product sizes were 350 bps from the wildtype and 450 bp from the JAM-C neo allele, respectively). Amplification with the primers 5′ -GGGAAGTCGCAAAGTTGTGAGTT- 3′ and 5′ -GCTCTAGACAGTGTTGCCGTCT- 3′ resulted in a product of 520 bps, in case mice were transgenic for the Tie2 JAM-C construct.

### Antibodies

For immunofluorescence stainings the following primary antibodies were used: 10 µg/ml polyclonal goat anti-mouse JAM-C (R&D systems, USA), polyclonal rabbit anti-JAM-C 526 (diluted 1/1000) and 1 µg/ml affinity-purified 526 [13], 10 µg/ml rat monoclonal anti-JAM-C (clone H33, from B. Imhof, Geneva, Switzerland [2]), undiluted monoclonal rat anti-mouse PECAM-1 hybridoma supernatants (clone Mec13.3 obtained from ATCC, USA; clone GC5.1 from B. Imhof [20]), 0.25 µg/ml polyclonal rabbit anti-human fibronectin that cross-reacts with mouse fibronectin and anti glial fibrillary acidic protein (GFAP) (DAKO Z0334, Denmark), and as isotype controls 10 µg/ml normal goat IgG, 10 µg/ml normal rabbit IgG (both R&D systems) and 5 µg/ml rat IgG2b (BD Bioscience, Switzerland). Additional antibodies used according to the recommendation of the manufacturer were: anti-neurofilament (NF; Sigma-Aldrich, USA), neuron-specific class III beta-tubulin (Tuj1; Covance, USA), rat anti-myelin basic protein (Millipore MAB386). Secondary antibodies were 3 µg/ml Cy3-conjugated donkey anti-goat IgG, 3 µg/ml Cy3-conjugated goat anti-rabbit IgG, 3 µg/ml Cy3-conjugated goat anti-rat IgG and 10 µg/ml AlexaFluor488-conjugated goat anti-rat IgG (all from Jackson ImmunoResearch Laboratories, Switzerland). For detection of endogenous mouse IgG by immunohistochemistry, 10 µg/ml biotinylated goat-anti mouse IgG was used.

### Immunofluorescence Analysis

Preparation of mouse tissue and staining procedures were performed mainly like published previously [21]. Briefly, mice were perfused with PBS or 1% formaldehyde in PBS through the left ventricle of the heart. Dissected tissues were embedded in Tissue-Tek (Sakura, Netherlands), frozen and stored at −80°C. For immunofluorescence stainings 6 µm cryostat sections were fixed in ethanol for 10 min at 4°C, followed by acetone for 1 min at room temperature with the exception to immunostaining for MBP, which required a fixation of 4% PFA/PBS for 10 min at room temperature. Blocking solution was composed of 10% normal rabbit serum in Tris-buffered saline (TBS). Primary antibodies were diluted in blocking solution and secondary antibodies in TBS with 10% normal mouse serum. Antibody incubation was performed for 1 h at room temperature. Sections were washed with TBS between incubation steps and finally mounted with Mowiol (Sigma-Aldrich, USA).

### Immunohistochemistry and Histology

For immunohistochemistry 6 µm cryostat sections were fixed in aceton for 10 min at −20°C, followed by blocking with 100% normal rabbit serum (AbD Serotec, Germany). Subsequently biotinylated primary antibody (diluted in PBS/0.5% BSA) and horseradish peroxidase-conjugated Streptavidin (Vector Laboratories, USA) were incubated with the sections for 30 min each. In between, sections were washed with PBS and PBS/0.1% Tween20. Sections were then developed with AEC substrate from Vectastain ABC Kit for 10 min (Vector Laboratories, USA), counterstained with hemalum (Merck, Germany) and coverslipped with Aquatex (Merck, Germany).

For GFAP and NF/Tuj1 staining, 3 µm paraffin sections were dewaxed and antigen retrieval was carried out in citrate buffer (pH 6.0). Blocking was either performed with normal goat serum (NGS) for GFAP or with the mouse-on-mouse (MOM, Vector Laboratories, USA) kit for NF/Tuj1, according to the manufacturers’ protocol. Subsequently, appropriate horseradish peroxidase-conjugated secondary antibodies were incubated with the sections for 1 h, followed by washes with PBS. Sections were then developed with AEC substrate from Vectastain ABC Kit for 20 min (Vector Laboratories, USA), counterstained with hemalum (Merck, Germany) and coverslipped with Aquatex (Merck, Germany).

Neuropathological examination of the murine brains was performed in serial sections by classical HE stains as well as iron staining for the detection of elder hemorrhages.

### Ventricular DiI Injections

For intracranial DiI injection into the lateral ventricle, 8 to 10 weeks old mice were anesthetized by intraperitoneal injection of Ketamin/Xylazin according to approved experimental protocols (University of Frankfurt, Germany). Mice were inserted into a stereotactic device and injected into the left lateral ventricle at 0,0 mm anterior-posterior to bregma, 0.75 mm left to midline and 1,5 mm ventral from the dura, via a pre-drilled hole in the skull. The hole was sealed with dental wax and the skin was sewed. After 24 h of incubation mice were anesthetized by a sub-lethal intraperitoneal injection of Ketamin/Xylazin, followed by cardiac perfusion for 2 minutes with PBS and subsequently for 4 minutes with 4% PFA. Mouse brains were removed and incubated over night in 4% PFA at 4°C. The next day they were transferred in 0.1 M phosphate buffer pH 7.4 containing 30% sucrose and incubated for several days at 4°C until brains were totally infiltrated with the solution. Brains were embedded in OCT compound for 12 µm cryo-sections.

### Ethics Statement

This is not applicable to the present study.

## Results

### JAM-C^−/−^ C57BL/6 Mice Develop a Severe Hydrocephalus

**Figure 1:**
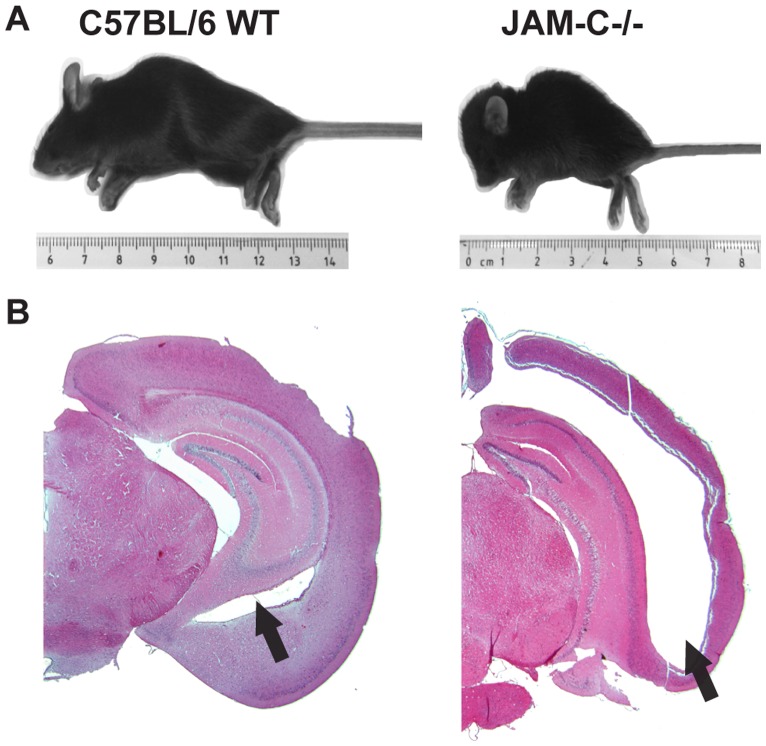
JAM-C^−/−^ C57BL/6 mice develop a severe hydrocephalus. A In comparison to age-matched C57BL/6 wildtype littermates JAM-C^−/−^ C57BL/6 mice are often smaller and display a hydrocephalus. **B** Histological analysis of hemalaun-stained brain cryosections reveals greatly enlarged lateral ventricles (arrows), a strikingly thinned cortex and overall alteration of brain anatomy in JAM-C^−/−^ C57BL/6 mice.

When backcrossing JAM-C^−/−^ mice into the C57BL/6 background we found that JAM-C^−/−^ C57BL/6 mice developed a severe hydrocephalus. JAM-C^−/−^ mice, but not JAM-C^+/−^ or wildtype littermates presented with growth retardation accompanied by an extremely enlarged and deformed skull (Figure 1A). Immunohistological analysis of frozen sections of brain tissues revealed dramatically dilated lateral ventricles, a markedly thinned cortex and, due to the increased hydrostatic pressure, a dislocation of the hippocampus in JAM-C^−/−^ mice (Figure 1B and not shown).

Although JAM-C^−/−^ mice housed in individually ventilated cages were born at correct Mendelian ratios (25.4% JAM-C^−/−^; 47.8% JAM-C^+/−^ and 26.9% wildtype offspring of 67 animals genotyped between P4 and P11) only 50% of the JAM-C^−/−^ mice survived until weaning at postnatal day 21 and two thirds of those developed a hydrocephalus within the first 4 to 8 weeks after birth (Table 1). No significant gender-specific differences in survival of JAM-C^−/−^ mice or incidence of hydrocephalus formation were detectable (Table 1).

**Table 1:** Number of JAM-C^−/−^ C57BL/6 offspring and hydrocephalus incidence.

	Offspring at postnatal day 21*		Hydrocephalus (HC)	
	total number	% JAM-C−/− offspring (% expected)	JAM-C−/− pups with HC (%)	JAM-C+/− or WT pups with HC (%)
	147	12.2 (25.0)	66.7	0
f	77	6.1	77.0	0
m	70	6.1	55.6	0

JAM-C+/− mice were interbread and offspring was genotyped at postnatal day 21. Abbreviations: f = female offspring; m = male offspring.

**Figure 2:**
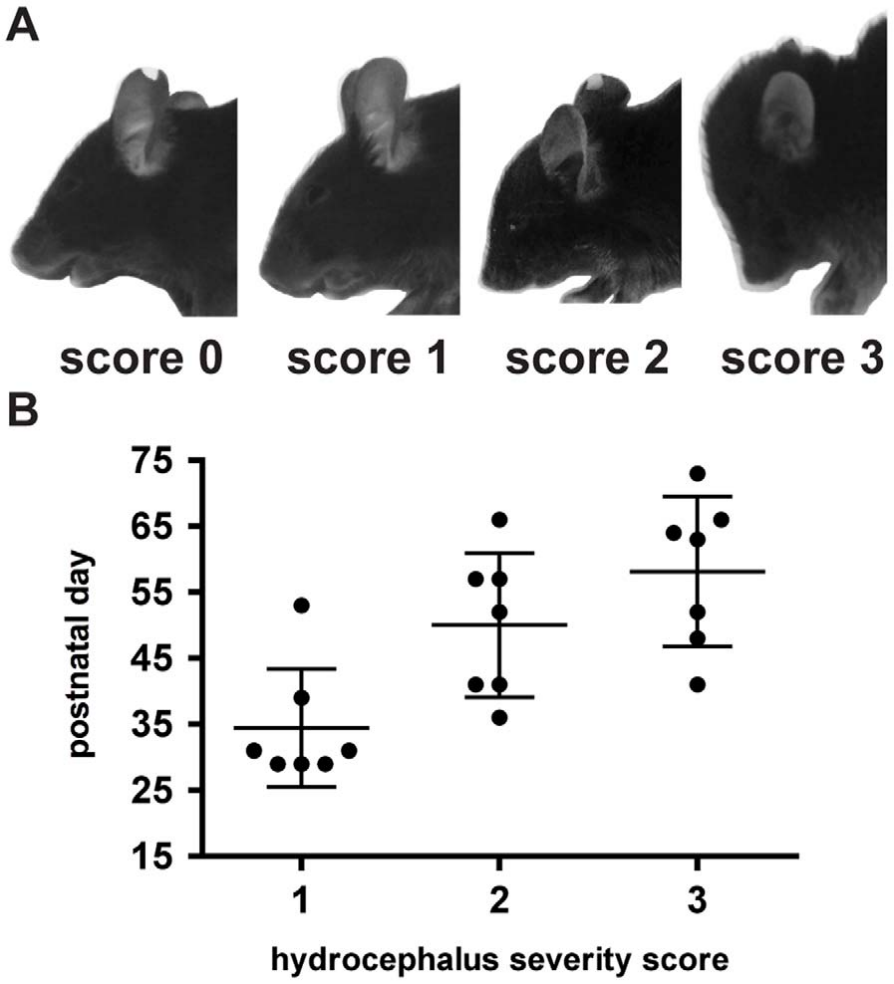
Hydrocephalus in JAM-C^−/−^ C57BL/6 mice develops over time. A: Different stages in hydrocephalus severity of JAM-C^−/−^ C57BL/6 mice are shown with their assigned scores. **B:** Seven male JAM-C^−/−^ C57BL/6 mice were scored weekly for changes in hydrocephalus severity. On average, first hydrocephalus symptoms appeared around 1 month after birth. Score 2 was reached about 2 weeks later. Finally, score 3 developed in most mice within the following week.

To investigate the development of the hydrocephalus over time in more detail, we developed a visual scoring scale evaluating the severity of the hydrocephalus as follows: 0 = macroscopically normal phenotype; 1 = normal snout, slightly enlarged skull; 2 = pointed snout, enlarged skull, often untended coat; 3 = extremely pointed snout, extremely enlarged skull (Figure 2A). The increase in hydrocephalus severity of seven JAM-C^−/−^ males was in depth monitored on a daily basis over a period of two months, starting at weaning with mice that had not yet developed a macroscopic hydrocephalus (Figure 2B). A slightly enlarged skull (score 1) was first detected between 4 to 8 weeks of age. On average, 16 days after scoring 1, mice developed a score 2 and eight days later a score 3. Additionally, we observed that a severe hydrocephalus (score 3) was accompanied by an aggravation of the general health conditions of these animals. Mice displayed a spastic gait, lethargy, tremor and untended coat and died within several days. Therefore, all further mice were sacrificed as soon as they reached score 3.

Taken together, when backcrossed into the C57BL/6 background, JAM-C^−/−^ mice developed a hydrocephalus within the first weeks of life. Severity of this phenotype increased over time and eventually led to the early death of these animals.

### JAM-C is Expressed in the CNS of C57BL/6 Mice

**Figure 3:**
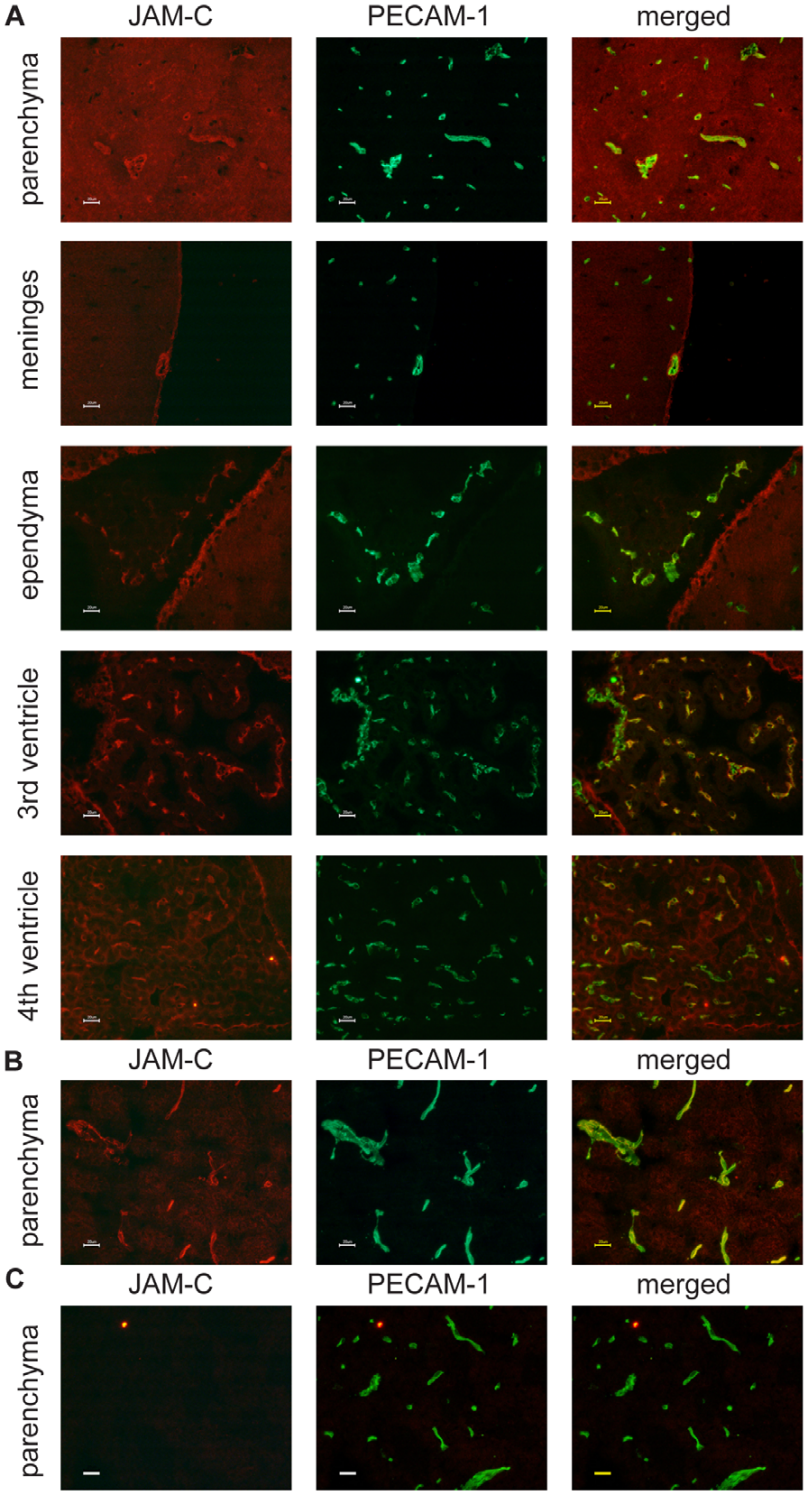
Expression of JAM-C in the mouse brain. Expression of JAM-C (red) in the brain was detected via indirect immunofluorescence staining of brain cryosections. Counterstaining for PECAM-1 (green) was performed to identify the vasculature. **A** In C57BL/6 wildtype mice JAM-C was localized to parenchymal blood vessels, meningeal vessels, the ependyma, the choroid plexus endothelium and the choroid plexus epithelium of the 4^th^ ventricle. Distinct junctional JAM-C staining was found in the endothelial cells of brain parenchymal vessels of Tie2 JAM-C brain tissues (**B**), while none was detectable in the brain of JAM-C^−/−^ C57BL/6 mice (**C**). Bars = 20 µm.

As hydrocephalus development in JAM-C^−/−^ mice C57BL/6 mice strongly suggested the expression of JAM-C in the CNS, we performed double-immunofluorescence stainings for JAM-C and PECAM-1 on brain cryosections from wildtype and JAM-C^−/−^ C57BL/6 mice. In addition, we analyzed transgenic 57BL/6 mice overexpressing JAM-C in endothelial cells (Tie2 JAM-C mice). Comparing different tissue fixation protocols and anti-JAM-C antibodies available to us (see Material and Methods), we found reproducible and specific staining for JAM-C in the brain using a commercially available polyclonal goat anti mouse JAM-C antibody (R&D). JAM-C immunostaining was observed on PECAM-1^+^ endothelial cells of parenchymal and meningeal brain vessels, but also on endothelial cells of fenestrated microvessels within the choroid plexus (Figure 3A). Additional JAM-C immunostaining was detected on the choroid plexus epithelium of the 4^th^ ventricle and on ependymal cells lining the ventricles (Figure 3A). As expected, intense JAM-C staining was observed in the brain vasculature of Tie2 JAM-C mice, with primary localization to endothelial cell contacts (Figure 3B). No specific signals were detectable with an isotype control antibody (not shown) or when JAM-C^−/−^ brain sections were stained with anti-mouse JAM-C antibodies (Figure 3C).

**Figure 4:**
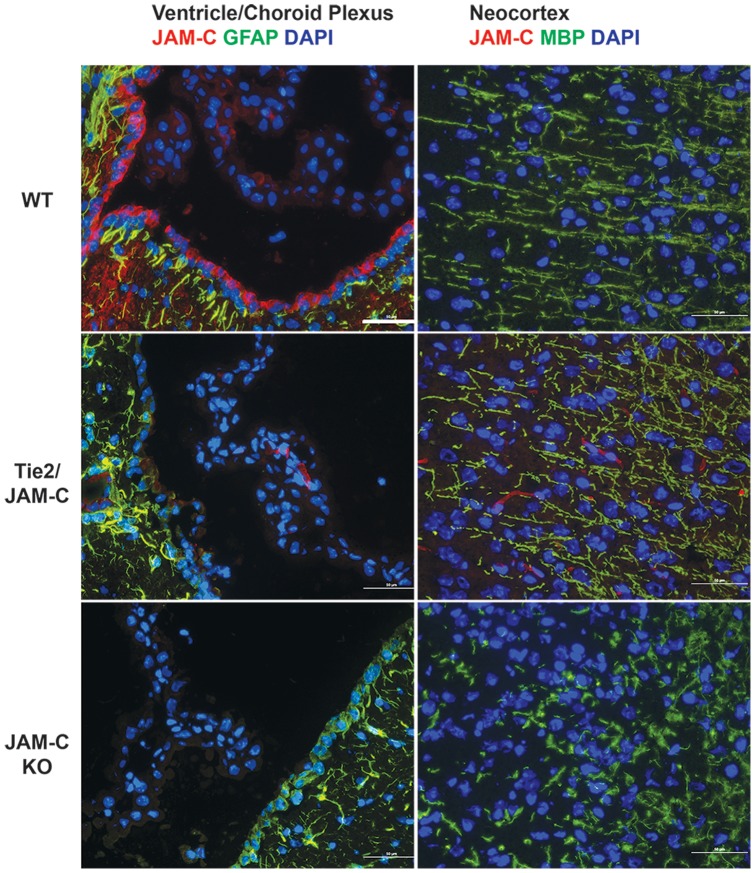
Expression of JAM-C in the mouse brain. Expression of JAM-C (red) in the brain was detected via indirect immunofluorescence staining of brain cryosections. Counterstaining for GFAP (green) and MBP (green) was performed to identify astrocytes and oligodendrocytes, respectively. JAM-C immunostaining did not co-localize with GFAP^+^ astrocytes or MBP^+^ oligodendrocytes in C57BL/6 wildtype mice (WT) and Tie2 JAM-C mice. Lack of detection of JAM-C immunostaining on blood vessels in WT but not Tie2 JAM-C mice is due to the necessity of using a fixation protocol for identifying MBP^+^ oligodendrocytes that is suboptimal for detection of JAM-C. Note that in the neocortex of JAM-C^−/−^ C57BL/6 mice, no MBP^+^ processes running perpendicular to the brain surface are visible. Bars = 50 µm.

Furthermore, we did not detect JAM-C immunostaining on glial fibrillary acidic protein (GFAP)^+^ astrocytes (Figure 4). Using the fixation protocol required to identify myelin-basic protein (MBP)^+^ oligodendrocytes we failed to see any JAM-C immunostaining on oligodendrocytes (Figure 4). As this fixation protocol is suboptimal for the detection of JAM-C we cannot exclude expression of JAM-C on oligodendrocytes, which has been described before [22], [23].

Taken together, expression of JAM-C was detectable on a variety of cells in the CNS and was not limited to CNS endothelium.

### Hydrocephalus in JAM-C^−/−^ C57BL/6 Mice is Accompanied with Reactive Gliosis and Defects in Cortex Lamination

**Figure 5:**
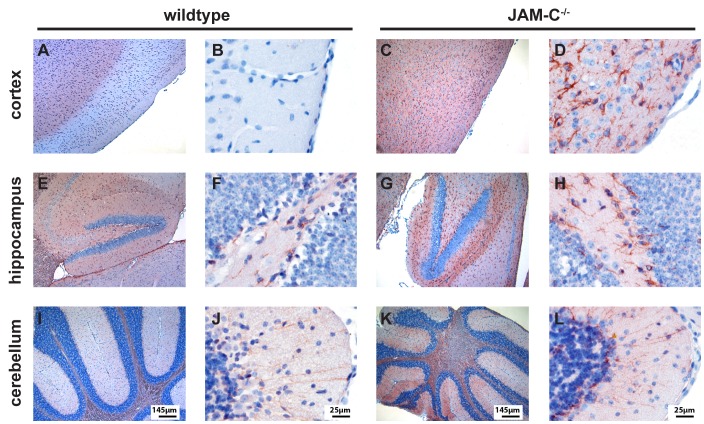
Hydrocephalus in JAM-C^−/−^ C57BL/6 mice is accompanied by reactive gliosis. Immunostaining for glial fibrillary acidic protein (GFAP) reveals reactive gliosis in JAM-C^−/−^ C57BL/6 mice. JAM-C^−/−^ C57BL/6 mice (symptomatic hydrocephalus score 1 shown here) exhibit extensive gliosis in the cortex (**4C** and **D**) and hippocampus (**4G** and **H**) as compared to wild-type littermates (**4 A, B, E** and **F**). In the cerebellum of JAM-C^−/−^ C57BL/6 only a mild increase of GFAP staining could be observed (**4 I** and **J**) as compared to wild-type cerebellum (**4 K** and **L**). Bars as indicated.

To evaluate in more detail the pathological alterations accompanied by the development of the hydrocephalus in JAM-C^−/−^ C57BL/6 mice we performed immunohistochemical stainings for glial fibrillary acidic protein (GFAP), comparing brains from JAM-C^−/−^ C57BL/6 mice with a hydrocephalus score of 1 and 2 with wild-type brains. A large number of reactive astrocytes with strong GFAP staining was observed in the anterior and posterior areas of the cortex as well as in the hippocampus of JAM-C^−/−^ C57BL/6 mice (Figure 4 and 5A–H). In contrast, the atrophic cerebellum of JAM-C^−/−^ C57BL/6 mice did not show any significant increase in GFAP^+^ astrocytes when compared to wild-type control cerebellum (Figure 5I–L).

**Figure 6:**
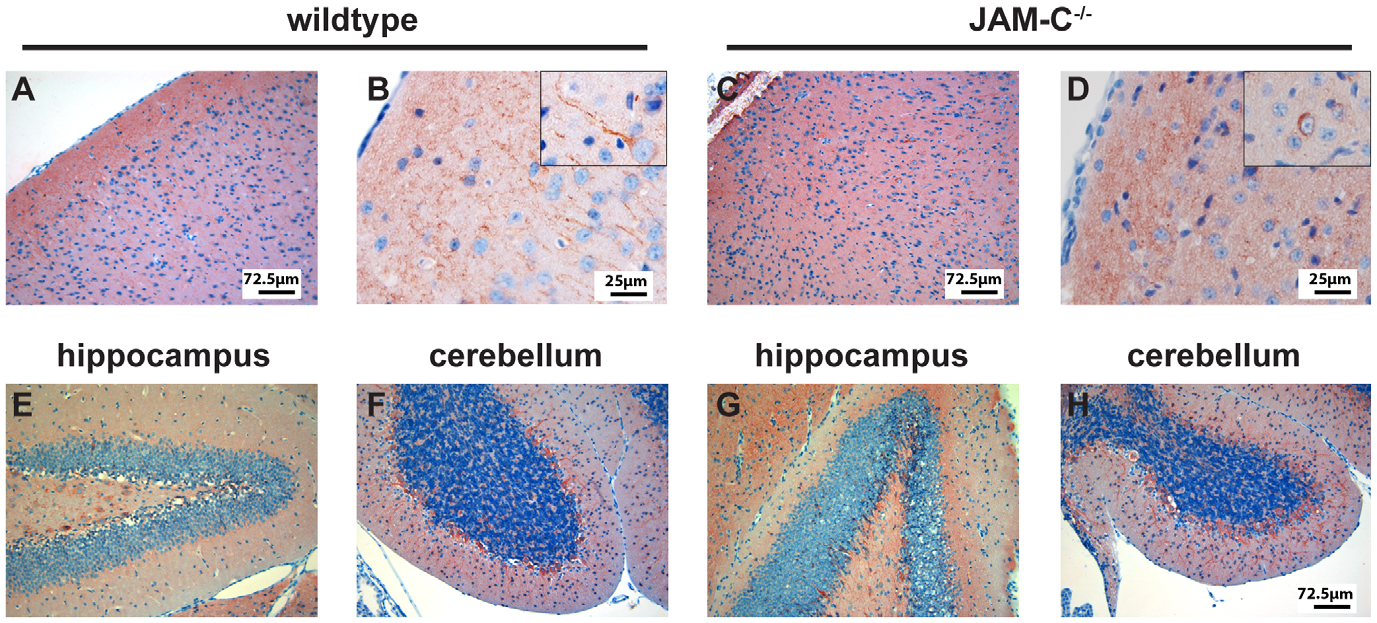
Hydrocephalus in JAM-C^−/−^ C57BL/6 mice is accompanied by loss of cortical layering. Immunostaining for neurofilament (NF) together with Tuj1 reveals loss of cortical layering in JAM-C^−/−^ C57BL/6 mice (hydrocephalus score 1 shown; **5 C** and **D**) when compared to wild type littermates (**5 A** and **B**). Inserts in **B** and **D** show pyramidal cells. Note that in the JAM-C^−/−^ C57BL/6 mice no axonal and dendritic processes are visible. In contrast, the overall structure of the hippocampus (**5 E** and **G**) and the cerebellum (**5 F** and **H**) of JAM-C^−/−^ C57BL/6 mice did not show any detectable alterations in NF/Tuj1 positive cells when compared to wild-type littermates. Bars as indicated.

By staining for the pan-neuronal markers neurofilament (NF) and the neuron-specific class III beta-tubulin (Tuj1) we observed a severe disorganization of cortical layers in brains of JAM-C^−/−^ C57BL/6 mice, including a lack of differentiated pyramidal cells (Figure 6A–D). In particular, no axonal and dendritic processes (Figure 6A–D) or MBP^+^ fibers (Figure 4) could be seen in brains of JAM-C^−/−^ C57BL/6 mice that ran perpendicular to the surface of the brain. The hippocampus as well as the cerebellum did not show any altered neuronal staining pattern (Figure 6E–H).

### Hydrocephalus in JAM-C^−/−^ C57BL/6 Mice is Accompanied by Hemorrhages

**Figure 7:**
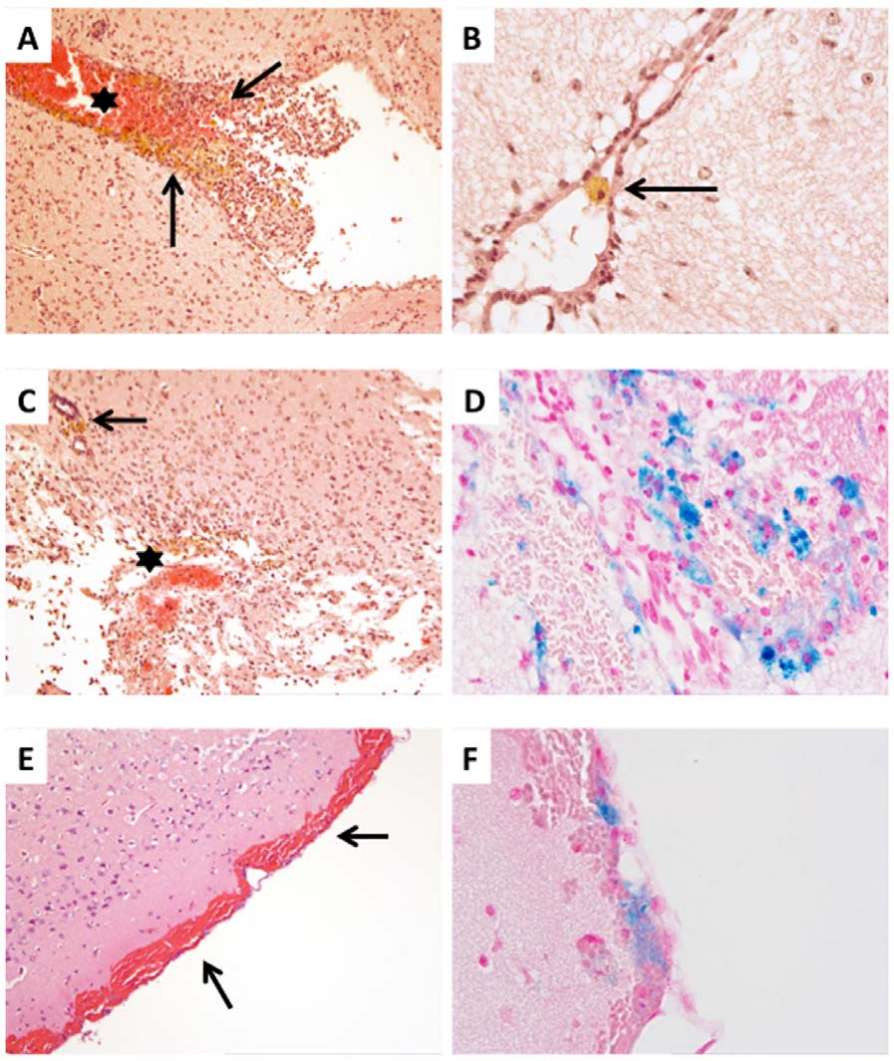
Hydrocephalus in JAM-C^−/−^ C57BL/6 mice is accompanied by hemorrhages. **A:** Hematoxylin and eosin staining showing signs of fresh (asterisk) and elder (arrows) bleedings within the III. ventricle. Elder bleedings consisted of erythrophages, hemosiderophages and also haematoidin pigment, the latter indicating bleedings older than 8 days (original magnification: 10x). **B:** Signs of elder bleedings were also observed within the aqueduct (arrow indicating hemosiderophage; HE stain; original magnification: 40x). **C:** Fresh and elder hemorrhages were also seen in the perivascular spaces (arrow) and within the CNS tissue (asterisk) (HE stain; original magnification 10x). **D:** Iron staining indicating the presence of abundant intraparenchymal iron-loaden macrophages (original magnification: 40x). **E, F:** Subarachnoidal hemorrhages presenting with areas of fresh (E; arrows; HE staining; original magnification: 10x) and elder bleedings (F; iron staining; original magnification: 40x).

As a loss of function mutation of JAM-C in humans was found to lead to severe hemorrhagic destruction of the brain [19], we next asked if the development of the hydrocephalus in JAM-C^−/−^ C57BL/6 mice was due to impaired cerebrovascular integrity and subsequent bleedings into the brain. Brain tissue sections of JAM-C^−/−^ C57BL/6 mice with a hydrocephalus score of 0, 1 and 2 and healthy wild-type C57BL/6 mice were stained with hematoxylin and eosin to detect tissue localization of erythrocytes. Additionally, we performed iron stainings to detect elder hemorrhages. This revealed fresh, subacute and old intraventricular, subependymal intraparenchymal as well as subarachnoid hemorrhages determined by the presence of vital erythrocytes, erythrophages, hemosiderophages and haematoidin pigment, respectively, in the brains of JAM-C^−/−^ C57BL/6 mice with a hydrocephalus but not in brains of JAM-C^−/−^ C57BL/6 mice without a hydrocephalus or in wild-type control mice (Figure 6). In addition, multiple perivascular bleedings were observed in the brain parenchyma solely in JAM-C^−/−^ C57BL/6 mice with a hydrocephalus but were absent in the brains of JAM-C^−/−^ C57BL/6 mice without a hydrocephalus and in wild-type C57BL/6 mice (Figure 7). These observations suggest that absence of JAM-C in the brain might lead to vascular disruption and subsequent bleedings, which could be causative for the development of the hydrocephalus. However, this would not rule out secondary bleedings related to increased intracranial pressure in mice with hydrocephalus or development of a hydrocephalus as a consequence of malresorption of CSF due to subarachnoid hemorrhage.

### Hydrocephalus in JAM-C^−/−^ C57BL/6 Mice is not Caused by the Lack of Vascular JAM-C

**Figure 8:**
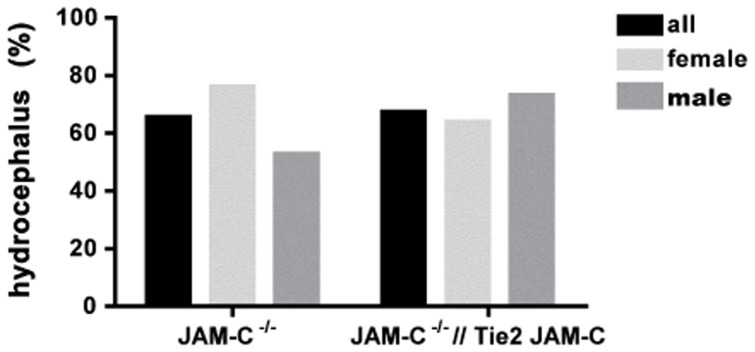
Endothelial re-expression of JAM-C does not rescue the hydrocephalus of JAM-C^−/−^ C57BL/6 mice. 38 JAM-C^−/−^ C57BL/6 mice (21 females and 17 males) and 40 JAM-C^−/−/^/Tie-2 JAM-C C57BL/6 mice (25 females and 15 males) were analyzed for hydrocephalus development. In comparison to JAM-C^−/−^ C57BL/6 mice no major difference in the hydrocephalus incidence could be observed when the expression of endothelial JAM-C was rescued in JAM-C^−/−^ C57BL/6 littermates.

In order to determine if the lack of endothelial JAM-C expression in the brain of C57BL/6 mice caused the development of the hydrocephalus, we bred JAM-C^+/−^//Tie2-JAM-C mice with JAM-C^+/−^ mice and compared the occurrence of the hydrocephalus and the survival of JAM-C^−/−^ versus JAM-C^−/−^//Tie2 JAM-C offspring. If all genotypes were produced at Mendelian ratios, i.e. if survival was unaffected by genotype, 12.5% JAM-C^−/−^ and 12.5% JAM-C^−/−^//Tie2 JAM-C offspring would be expected from these breedings. However, as shown in Table 2, the percentage of offspring that survived until weaning was reduced similarly for both of these genotypes (7.1% JAM-C^−/−^ and 8.2% JAM-C^−/−^//Tie2 JAM-C mice). Furthermore, the hydrocephalus incidence of about 65% was not significantly reduced in JAM-C^−/−^//Tie2 JAM-C as compared to JAM-C^−/−^ mice, with no major differences between male and female mice (Figure 8). Excluding the possibility that brain endothelial re-expression of JAM-C was incomplete in JAM-C^−/−^//Tie2 JAM-C mice vascular expression of JAM-C was indistinguishable by immmunostaining in brains of Tie2 JAM-C and JAM-C^−/−^//Tie2 JAM-C mice (data not shown).

**Table 2:** Endothelial JAM-C fails to rescue survival of JAM-C−/− C57BL/6 mice.

	total number of pups*	% JAM-C−/− offspring(% expected)	% JAM-C−/−//Tie-2 JAM-C offspring(% expected)
	574	7.1 (12.5)	8.2 (12.5)
f	297	8.1 (12.5)	8.8 (12.5)
m	277	6.1 (12.5)	7.6 (12.5)

* JAM-C+/− and JA-C+/−//Tie-2 JAM-C mice were interbred and offspring was genotyped at postnatal day 21. Abbreviations: f = female offspring; m = male offspring.

Thus, endothelial re-expression of JAM-C failed to rescue the hydrocephalus phenotype of JAM-C^−/−^ C57BL/6 mice demonstrating that lack of JAM-C in the cerebrovascular endothelium is not the major cause of the development of a hydrocephalus in JAM-C^−/−^ C57BL/6 mice.

### Evaluation of CSF Circulation within the Ventricular System of JAM-C^−/−^ Mice

**Figure 9:**
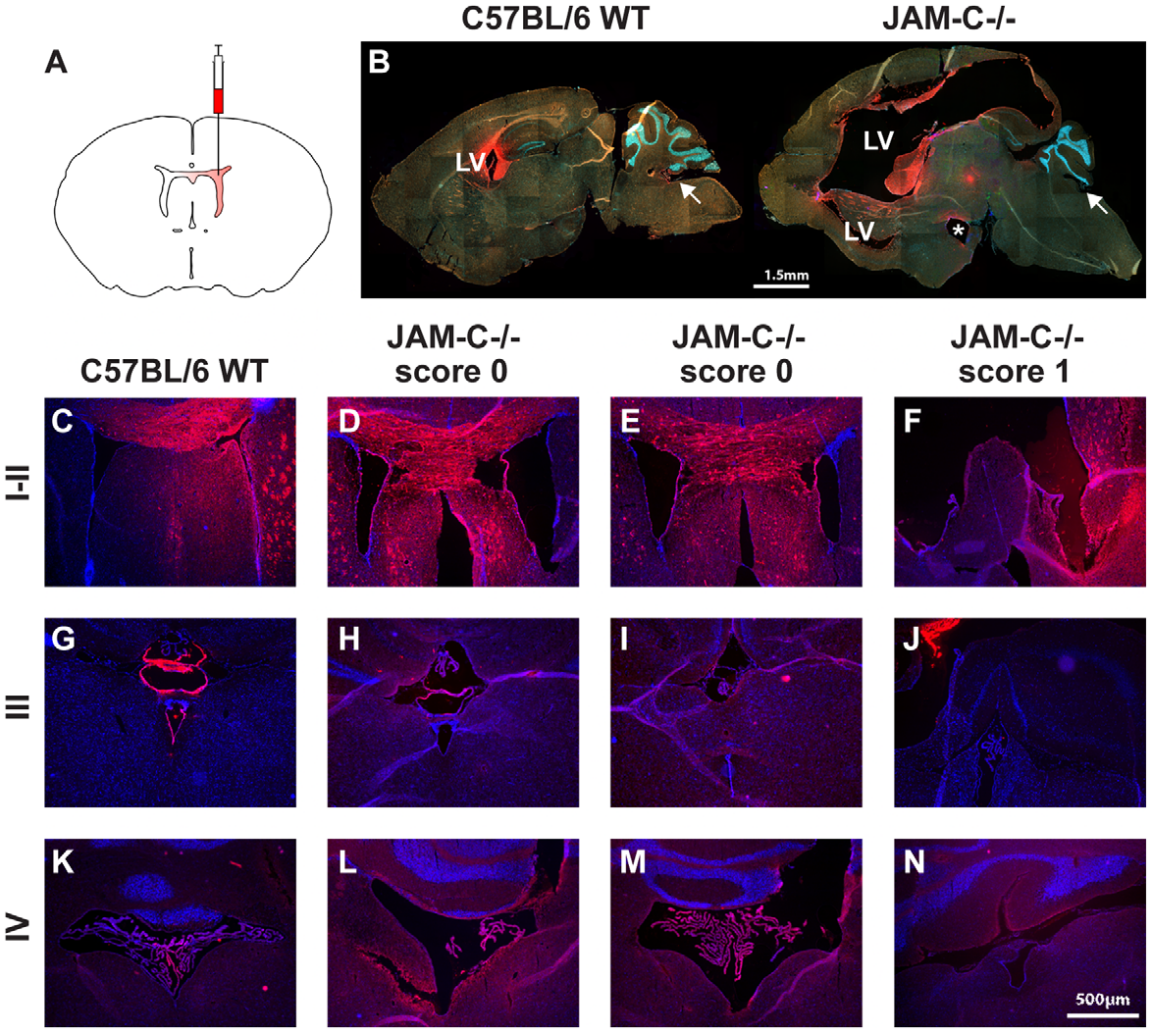
Lateral ventricle DiI injections do not reach the 4^th^ ventricle of JAM-C^−/−^ mice with symptomatic hydrocephalus. **A** Injection scheme for DiI is shown. **B** DiI (red) distribution in the ventricular system in sagittal sections from a C57BL/6 wild-type and a hydrocephalic JAM-C^−/−^ C57BL/6 mouse is shown. Nuclear staining (DAPI) is shown in blue, unspecific fluorescence in green. LV = lateral ventricle; asterisk = 3^rd^ ventricle; arrows point to 4^th^ ventricle. **C–F** DiI distribution within the lateral ventricles. **G–J** DiI distribution within the 3^rd^ ventricle. Note that in J (JAM-C^−/−^ score 1) the 3^rd^ ventricle contains little or no DiI. **K–N** DiI distribution within the 4^th^ ventricle. Note that in N (JAM-C^−/−^ score 1) the 4^th^ ventricle contains no DiI.

As we noticed an increasing severity of the hydrocephalus in individual mice over time, we investigated whether the enlargement of lateral ventricles could proceed and finally lead to occlusion of the cerebral aqueduct, a situation also observed in other hydrocephalus mouse models [24], [25]. To this end the fluorescent dye DiI was injected into the left lateral ventricle of wildtype and JAM-C^−/−^ mice to track CSF flow through the ventricular system (Figure 9A). Under healthy conditions, the dye follows the CSF movement from the lateral ventricles through the paired foramina of Monro into the 3^rd^ ventricle, followed by passage of the cerebral aqueduct and finally reaches the 4^th^ ventricle (for review see [26]). 24 hours after injection, mice were sacrificed and brains were frozen, sectioned and cell nuclei were stained with DAPI. Figure 8B shows DiI (red) distribution in the ventricular system in sagittal sections of a wildtype C57BL/6 and a JAM-C^−/−^ C57BL/6 mouse with pronounced hydrocephalus. In the wildtype mouse, DiI was detectable in the parenchyma around the left lateral and the 4^th^ ventricle (Figure 8B, left), while in the JAM-C^−/−^ C57BL/6 mouse the dye was distributed around the lateral ventricle, but was not visible in the displaced 3^rd^ and the 4^th^ ventricle. This strongly suggests that obstruction of the aquaduct cannot be the primary cause of the hydrocephalus (Figure 9B, right). To investigate possible differences between several graduations of hydrocephalus severity, brain coronal sections of two DiI-injected JAM-C^−/−^ C57BL/6 mice with score 0 and one JAM-C^−/−^ C57BL/6 mouse with score 1 were analyzed and compared to a C57BL/6 wildtype littermate (Fig. 9C–N). Unfortunately, injections of mice with more severe hydrocephalus (score 2–3) were not reliable, due to high intracranial pressure that pushed CSF and DiI out of the injection channel and because animals died during or shortly after the procedure. However, already in JAM-C^−/−^ mice with moderate hydrocephalus, no DiI was detectable around the 4^th^ ventricle (Figure 9B) or even 3^rd^ ventricle (Figure 9J). This is in contrast to the C57BL/6 wildtype control and the two JAM-C^−/−^ C57BL/6 mice with score 0, in which DiI was found in and around the injected lateral, the 3^rd^ and the 4^th^ ventricles (Figures 9C–E, G–I and K–M). In conclusion, those JAM-C^−/−^ C57BL/6 mice developing a hydrocephalus had acquired impaired CSF drainage from the lateral to the 3^rd^ ventricle probably due to the occlusion of the foramina of Monro or due to generally reduced CSF drainage caused by subarachnoid hemorrhage.

## Discussion

The present study extends our knowledge on the expression pattern of JAM-C in the central nervous system (CNS) and underlines an important role of JAM-C in brain homeostasis that can be modeled in JAM-C^−/−^ C57BL/6 mice, which is strikingly similar to the recently observed pathology in humans with a loss-of-function mutation in JAM-C [19].

With the aim to obtain JAM-C^−/−^ mice in a genetic background that is useful for studying a variety of neurological disorders we backcrossed JAM-C^−/−^ mice onto the genetic background of C57BL/6 mice. As previously reported for JAM-C^−/−^ mice with a mixed 129Sv - C57BL/6 background [15], JAM-C^−/−^ C57BL/6 mice were born in correct Mendelian ratios, but then showed growth retardation and reduced survival. Unexpectedly, more than 60% of JAM-C^−/−^ C57BL/6 mice that survived until weaning (but not their heterozygous or wildtype littermates) developed a hydrocephalus that increased in severity over time and finally led to the early death of the affected mice.

We therefore examined the expression of JAM-C in the brains of C57BL/6 mice by performing extensive immunofluorescence stainings using a panel of anti-JAM-C antibodies and by comparing brain sections from C57BL/6 mice, JAM-C^−/−^ C57BL/6 mice as well as from C57BL/6 mice overexpressing endothelial JAM-C (Tie-2-JAM-C mice). Using this approach we detected JAM-C specific immunofluorescence staining on endothelial cells of brain parenchymal and meningeal blood vessels as well as on endothelial cells of the fenestrated choroid plexus microvessels. Additionally, JAM-C immunostaining was seen on ependymal cells lining the ventricular system and on the choroid plexus epithelium of the 4^th^ ventricle. Our findings are in line with recent obervations detecting JAM-C specific immunostaining in mouse brain endothelial and ependymal cells [22] [23]. In addition these studies assigned expression of JAM-C to oligodendrocytes and neural stem cells. In apparent contast to these recent findings JAM-C has originally been reported to be absent in the mouse brain [17], [27]. We speculate that this might be due to different antibodies used, as also in our hands not all tested anti-JAM-C antibodies performed well on the highly myelinated CNS tissue. Differences in tissue fixation and thus epitope exposure as experienced by us might additionally lead to lack of JAM-C immunoreactivity within the CNS described in those earlier studies.

Hydrocephalus is a consequence of net cerebrospinal fluid (CSF) accumulation in the CNS and has multiple causes. According to the classical view there is either impairment of CSF flow within the ventricular system (non-communicating hydrocephalus) or disturbed absorption of CSF (for review see [26]). In addition dysplasia of the cerebral cortex and neuronal degeneration due to several other reasons appear to be implicated in hydrocephalus pathogenesis [28]–[30]. In mammals the generally accepted model of CSF circulation assumes a bulk flow of choroid plexus-produced CSF from the lateral ventricles to the 3^rd^ and 4^th^ ventricle and finally into the subarachnoid space, where it is absorbed mainly by arachnoid villi (reviewed in [26]). Nevertheless, the drainage of CSF through BBB capillaries is discussed as well (reviewed in [31]).

Depending on the cell type and experimental condition JAM-C has been shown to either enhance or reduce paracellular permeability [2], [27], [32]–[34]. JAM-C might therefore be implicated in regulating BBB integrity or the absorption of CSF through the BBB. Dysfunction of the brain vasculature in JAM-C^−/−^ C57BL/6 mice was suggested by the observation of perivascular and subarachoidal hemorrhages. Therefore we attempted to rescue endothelial JAM-C expression by introducing the Tie2-JAM-C transgenic line into the JAM-C^−/−^ background. Yet, neither mortality nor hydrocephalus incidence were reduced in the resulting JAM-C^−/−^//Tie2 JAM-C mice. This finding indicates that hydrocephalus development in JAM-C^−/−^ C57BL/6 mice depends on non-endothelial JAM-C expression. This is further underlined by the recent observations that C57BL/6 mice with endothelial-cell specific deletion of JAM-C neither show a high mortality rate nor development of a hydrocephalus [35]. Genetic background also plays a key role, as hydrocephalus development was not observed in JAM-C^−/−^ mice in a genetically mixed background [9], [15], [17]. The latter might be due to unknown modifier genes that influence the effects of JAM-C absence, as reported for other mutant mouse models that also presented a hydrocephalus solely in the C57BL/6 background [29], [36]–[39].

Occlusion of the cerebral aqueduct is a widely accepted cause of hydrocephalus, but is also observed secondary to ventricular dilation [26]. To test if the hydrocephalus in JAM-C^−/−^ C57BL/6 mice displayed aqueduct occlusion we injected the fluorescent tracer DiI into the left lateral ventricle of wildtype and JAM-C^−/−^ C57BL/6 mice and evaluated its distribution within the ventricular system. As expected, in wildtype and non-hydrocephalic JAM-C^−/−^ C57BL/6 mice DiI was detectable in and around the lateral, 3^rd^ and 4^th^ ventricles, while in JAM-C^−/−^ C57BL/6 mice with symptomatic hydrocephalus the dye was prevented to reach the 4^th^ or even the 3^rd^ ventricle. By performing histology on serial sections of two hydrocephalic JAM-C^−/−^ and two C57BL/6 wildtype mice we additionally failed to show any malformation of the cerebral aqueduct (not shown). Thus, occlusion of the aqueduct is not the cause of the development of a hydrocephalus in JAM-C^−/−^ C57BL/6 mice. Rather hydrocephalic JAM-C^−/−^ C57BL/6 mice have acquired a reduced CSF drainage from the lateral to the 3^rd^ ventricle suggesting either a CSF drainage block at the level of the foramina of Monro or generally reduced CSF drainage in these mice that may be due to subarachnoid hemorrhage. Ependymal cells cover the walls of the ventricular system and their cilia movement seems essential for adequate CSF flow [40]–[42]. Moreover, absence or disruption of proper cilia motility is associated with hydrocephalus in men and rodents [43]–[45]. JAM-C expressed in ependymal cells as found by us in the present study and others before [22], [23] might be involved in regulating ependymal polarization and thus permeability of these CSF facing epithelial layers [9], [12].

An alternative or complementary reason for the development of a hydrocephalus in JAM-C^−/−^ C57BL/6 mice might be due to JAM-C expression in CNS parenchmyal cells. The Src-family tyrosine kinase Fyn has been reported to be essential for oligodendrocyte development and Fyn-deficiency was shown to lead to cortical thinning, axon degeneration and subsequent severe hydrocephalus [29]. Given the fact that JAM-C was recently found in human oligodendrocytes and was suggested to act upstream of c-Src [22], combined with our observation of JAM-C expression in the cerebral cortex, loss of cortical layering and cortical thinning in hydrocephalic JAM-C^−/−^ C57BL/6 mice, JAM-C could be implicated in the above described pathogenesis.

Additionally, JAM-C has been observed in neural stem cells in the developing and adult brain and has been suggested to mediate asymmetric cell division and to maintain the brain stem cell compartment by inhibiting neural stem cell migration into deeper cortical layers [23]. JAM-C expressed by cerebellar granule neurons was shown to be essential for their migration out of germinal zones during post-natal brain morphogenesis by recruiting JAM-C to the neuronal surface [46]. Combined with the previous observation that impaired migration of cortical neurons can induce congenital hydrocephalus [28] these observations allow to speculate on an alternative etiology of hydrocephalus development in JAM-C^−/−^ C57BL/6 mice.

In contrast to occlusion of CSF flow, the development of a hydrocephalus may be the result of CSF overproduction linked to choroid plexus papilloma or diffuse villous hyperplasia of the choroid plexus (for review see [47], [48]). As we never observed any changes in choroid plexus morphology in hydrocephalic JAM-C^−/−^ C57BL/6 mice we consider it less likely that the absence of JAM-C in the 4^th^ ventricle choroid plexus epithelium is implicated in hydrocephalus initiation by overproduction of CSF.

Taken together, the present study further confirms JAM-C expression in the brain of mice, more precisely the BBB and choroid plexus endothelial cells, the epithelial cells of the 4^th^ ventricle choroid plexus, and the ependymal cells lining the ventricles. JAM-C^−/−^ mice in the C57BL/6 genetic background developed a severe and progressive hydrocephalus, characterized by extremely enlarged lateral ventricles accompanied by hippocampal dislocation, cortical thinning, loss of proper cortical layering with subsequent reactive gliosis as well as hemorrhages in different brain compartments and obstruction of CSF circulation. In contrast to JAM-C^−/−^ mice in mixed 129Sv - C57BL/6 background, JAM-C^−/−^ C57BL/6 mice therefore mimic part of the CNS pathology recently observed in members a large consanguineous family carrying a rare mutation in JAM-C leading to a non-functional protein [19]. Affected individuals develop a syndrome characterized by severe hemorrhagic destruction of the brain, sub-ependymal calcifications, enlarged ventricles and congenital cataracts partially reflected in JAM-C^−/−^ C57BL/6 mice. As endothelial re-expression of JAM-C failed to increase survival or inhibit hydrocephalus formation in JAM-C^−/−^//Tie2 JAM-C mice our observations point to non-endothelial JAM-C to be important for CNS homeostasis, although further investigations are required to elucidate its exact role in hydrocephalus pathogenesis.
